# The Conformational Structural Change of Soy Glycinin via Lactic Acid Bacteria Fermentation Reduced Immunoglobulin E Reactivity

**DOI:** 10.3390/foods10122969

**Published:** 2021-12-02

**Authors:** Zhen Liu, Yaqiong Wang, Yifei Liu, Qiuqin Zhang, Wei Li, Mingsheng Dong, Xin Rui

**Affiliations:** College of Food Science and Technology, Nanjing Agricultural University, Nanjing 210095, China; Liuzhenlllz@163.com (Z.L.); 2020108038@njau.edu.cn (Y.W.); 2019108008@njau.edu.cn (Y.L.); zqq@njau.edu.cn (Q.Z.); lw1981@njau.edu.cn (W.L.); dongms@njau.edu.cn (M.D.)

**Keywords:** glycinin, lactic acid bacterial, IgE reactivity, particle size distribution, FTIR, intrinsic fluorescence, interaction between proteins

## Abstract

This study investigated the fermentation of isolated soy glycinin by using the *Lactiplantibacillus* plantarum B1-6 strain, its reduction effect on immunoglobulin E (IgE) reactivity, the relationship with protein aggregation/gelation state and conformational changes. Fermentation was performed under different glycinin concentrations (0.1%, 0.5%, 1% and 2%, *w*/*v*) and varied fermentation terminal pH levels (FT-pH) (pH 6.0, 4.5, 4.0 and 3.5). *L. plantarum* B1-6 showed potency in reducing immunoreactivity to 0.10–69.85%, as determined by a sandwich enzyme-linked immunosorbent assay. At a FT-pH of 6.0 and 4.5, extremely low IgE reactivity (0.1–22.32%) was observed. Fermentation resulted in a great increase (2.31–6.8-fold) in particle size and a loss of intensity in A3 and basic subunits. The conformation of glycinin was altered, as demonstrated by improved surface hydrophobicity (1.33–7.39-fold), decreased intrinsic fluorescence intensity and the α-helix structure. Among the four selected concentrations, glycinin at 1% (*w*/*v*, G-1) evolved the greatest particles during fermentation and demonstrated the lowest immunoreactivity. Principal component analysis confirmed that particle size, intrinsic fluorescence intensity, α-helix and ionic bond were closely related to immunoreactivity reduction.

## 1. Introduction

Food allergy is a serious problem for food safety worldwide, as it poses a threat to the health and safety of allergy sufferers. Approximately 1–2% of adults and 8% of young children suffer from food allergies [1]. Soybean, which contains a high number of proteins (35–40%) and is a good source of high-quality proteins [2], belongs to the group of the ‘big eight’ allergens. Soy allergy has a prevalence of 0.1–3.2% and 0.1–5.7% in adults and children, respectively [3]. Allergenicity caused by soybean is mainly due to its proteins. Soy protein isolate (SPI), which is composed of at least 90% protein basis, contains several soybean allergens [4]. Predominant soybean allergens include the major storage proteins β-conglycinin (7S globulin) and glycinin (11S globulin) [5]. Glycinin, a protein composed of five different subunits, namely A1aB2, A2B1a, A1bB1b, A5A4B3 and A3B4, has been identified as one of the major allergens in soybean, and its antigenicity has been wildly reported [6,7]. A previous study showed that among 30 sera from soy allergic patients, 86% of subjects possessed specific IgE immunoreactivity to glycinin or β-conglycinin [6]. Another report demonstrated that glycinin triggered severe allergic symptoms among 74 subjects (range, 0.6–16.3 years) [8]. Kern et al. identified 405 potential immunologic epitopes from soy protein, and 60% of them were related to glycinin [9]. In addition, by using a three-dimensional (3D) model, nine possible epitopes were predicted, and most of them were exposed on the surface of soy glycinin [7]. Another study demonstrated that the immunogenicity of glycinin was mainly related to its acid subunit [10].

Several technologies have been developed to reduce the antigenicity of soy proteins, including thermal and non-thermal processing (e.g., high hydrostatic pressure treatment, enzymatic hydrolysis, genetic modification and microbial fermentation) [3,11,12,13,14]. Fermentation, an ancient processing approach, demonstrated improvements in the functional and nutritional properties of soy products [15]. Fermentation by lactic acid bacteria (LAB) was considered to have potential in reducing soy protein antigenicity [5]. A previous study demonstrated that *Lactiplantibacillus helveticus*, compared with *Bacillus subtilis* and *Rhizopus oryzae*, showed the strongest capacity in reducing soy protein immunoreactivity, up to 100% [16]. Another study demonstrated a 94.4% reduction in the immunoreactivity of fermented soymilk by using Enterococcus faecalis VB43 [11].

The potent capacity of LAB to reduce soy protein immunoreactivity has been considered to correspond to the ability to alter protein structure. Earlier studies suggested fermentation by LAB might induce the degradation of protein, further leading to a break-down of epitopes [11]. A more recent study reported that lactic fermentation significantly reduced the solubility of soy protein, thus suggesting changes in higher structures and aggregation states might be responsible for reducing immunoreactivity [16]. Our previous research demonstrated LAB was able to change secondary and tertiary structures of soy protein, and those changes could be contributed to IgE reactivity reduction [13]. These studies revealed the possibility that the reduction in soy immunoreactivity by LAB might be due to higher structure alternation. However, this hypothesis was mainly based on crude soy proteins. Proteins need to be fractionated in order to precisely identify the changes in their structure during LAB fermentation.

Therefore, glycinin was isolated in the current study. L. plantarum B1-6, a strain showing potential in reducing SPI immunoreactivity, was selected [13,17]. Fermentation was performed under different glycinin concentrations (0.1%, 0.5%, 1% and 2%, *w*/*v*) and varied fermentation terminal pH levels (FT–pH) (pH 6.0, 4.5, 4.0 and 3.5). IgE reactivity was monitored during fermentation. The protein aggregation and conformation were investigated by gelling behavior, SDS-PAGE, dynamic light scattering (DLS), Fourier transform infrared spectroscopy (FTIR), intrinsic fluorescence, and surface hydrophobicity. Interactions between proteins were also studied. The relationship between IgE reactivity and conformational changes was further discussed.

## 2. Materials and Methods

### 2.1. Materials, Chemicals, and Microorganism

Soybeans were obtained from a local supermarket in Nanjing, P. R. China and stored at 4 °C until use. Molecular mass standard was purchased from Sangon Biotech (Shanghai, China). All other chemical regents used were of analytical grade and obtained from Sigma-Aldrich (St. Louis, MO, USA).

*Lactiplantibacillus plantarum* B1-6 were isolated from Xinjiang Kirgiz boza, a traditional cereal-based drink in Xinjiang Vygur autonomous region of China. The strain was given the gene accession number KM200717.

### 2.2. Glycinin Extraction

Glycinin extraction was conducted following a previous protocol [12]. All centrifugation was performed at 10,000× *g*, 4 °C for 15 min in a centrifuge (TGL-16R, Shan Dong BaiO Medical Technology CO., LTD., Shandong, China). A pH meter (Testr30, Oakton, Singapore) was used to adjust pH during extraction by using HCl (2 mol/L) or NaOH (2 mol/L). Whole soybean seeds were grounded by a grinding mill (RS-FS1401, Royalstar, Anhui, P. R. China) and defatted in n-hexane. Defatted soy flour was then mixed with distilled water in a ratio of 1:15 (*m*/*v*), and pH was adjusted to 9.0. The slurry was subsequently incubated at 45 °C for 1 h before supernatant was separated by centrifugation. The pooled supernatants were added to NaHSO3 (0.01 mol/L) at an adjusted pH of 6.4 and maintained overnight at 4 °C. The solution was again centrifuged, and supernatant was discarded. The sediment, glycinin, was lyophilized.

### 2.3. Fermentation of Glycinin by L. plantarum B1-6

#### 2.3.1. Inoculum Preparation

*L. plantarum* B1-6 was activated in Man Rogosa and Sharp broth (MRS, pH 6.2). After two successive cultures in MRS broth at 37 °C, the bacteria were harvested by centrifugation at 7500× *g* for 10 min and washed twice with 0.85% (*w*/*v*) NaCl for further use.

#### 2.3.2. Lactic Fermentation

Lyophilized glycinin powder was dissolved in water at 0.1%, 0.5%, 1% and 2% (*w*/*v*) to prepare a series concentration of glycinin solution, and the pH was adjusted to 7.0 with 2 mol/L NaOH. After pasteurization at 85 °C for 10 min, sterilized glucose solution was added to achieve a final concentration of 2% (*w*/*v*). Inoculation ratio of L. plantarum B1-6 was 3% (*v*/*v*), and fermentation was conducted at 37 °C. A pH meter (Testr30, Oakton, Singapore) was used to constantly monitor pH change during fermentation. Fermentation was terminated when pH dropped to 7.0, 6.0, 4.5, 4.0 and 3.5, respectively. Fermentation was performed in duplicates.

### 2.4. Immunoassay

Immunoassay was measured by using a Sandwich enzyme-linked immunosorbent assay according to a previous study by using the RIDASCREEN^®^ FAST Soya ELISA test kit (R7102, R-Biopharm AG, Darmstadt, Germany) [18]. The concentration of samples was adjusted to 2.5–20 μg/mL. One hundred μL of sample was added to primary antibody-coated microtiter plate wells and incubated for 10 min at 25 °C. To remove excess unbound proteins, the wells were washed twice by PBST solution (pH 7.0, containing 0.05% Tween 20). One hundred μL of horseradish peroxidize-coupled secondary antibody was subsequently added and incubated for another 10 min at 25 °C. Wells were then washed again before addition of 100 μL of substrate solution to initiate the calorimetric reaction. The incubation was performed at 25 °C for 10 min in the dark, and the reaction was terminated by addition of 100 μL of stop solution. The absorbance was recorded at 450 nm by BioTek μQuant Microplate Spectrophotometer (BioTek Instruments, Inc., Winooski, VT, USA). The relative IgE reactivity (%) was calculated based on the following equation:

IgE reactivity (%) = OD450 nm (Sample, fermented glycinin)/OD450 nm (unfermented glycinin) × 100%(1)

### 2.5. Protein Gelation/Aggregation Assessments

#### 2.5.1. Gelling Properties

Gel-forming ability was analyzed by a previous protocol with minor modifications [19]. A firm gel was deemed to have been formed when the sample was inverted and the suspension did not flow. A weak gel was deemed to have been formed when a semi-solid was formed that flowed somewhat on inversion. The smallest gelling concentration (LGC) was estimated as the critical concentration below which no self-supporting gel was formed.

#### 2.5.2. Particle Size Distribution

Particle size distribution was measured by an integrated laser light-scattering instrument (Mastersizer 3000, Malvern Southborough, MA, USA). The refractive index of the water dispersant was set as 1.333, and refractive index of the scatterers of the sample solution was set as 1.46. The analysis was conducted in triplicate.

#### 2.5.3. Electrophoresis

Sodium dodecyl sulfate polyacrylamide gel electrophoresis (SDS-PAGE) was employed to analyze protein profiles of glycinin during fermentation. SDS-PAGE was carried out in a Bio-Rad Miniprotein 3 unit (Bio-Rad Laboratories, Inc., Hercules, CA, USA) with stacking gel at 4% and separating gel at 12%. SDS-PAGE was carried out under reducing conditions by supplying 5% β-ME. Running the gel at 80 V and 120 V for stacking and separating gel, respectively. A pre-stained broad range standard (15–130 kDa) was used. Gels were then scanned with Image Scanner III (GE Healthcare Biosciences, Uppsala, Sweden) and analyzed by using Quantity One software, version 4.6.2 (Bio-Rad Laboratories, Inc., Hercules, CA, USA).

### 2.6. Protein Conformation Assessments

#### 2.6.1. Surface Hydrophobicity

8-anilino-1-naphthalenesulfonic acid (ANS) was employed as a fluorescence probe to determine the surface hydrophobicity of the fermented samples according to a previous study [20]. Samples were diluted serially (0.2–1 mg/mL) with PBS (0.1 M, pH 7.6). A total of 5 μL ANS (8 mmol/L) was then added to 1 mL of prepared protein samples and protected from light for 20 min. Relative fluorescence intensity was recorded by a SpectraMax fluorescence spectrophotometer (Molecular Devices, San Jose, CA, USA) with excitation wavelengths of 390 nm and emission wavelengths of 470 nm. All intensity was corrected by subtraction of the corresponding sample without the addition of ANS. The initial slope of the intensity versus protein concentration was calculated by linear regression analysis with Microsoft Excel for Windows 10 (version 2019) and used as an indicator of the protein surface hydrophobicity. Three replicates were carried out.

#### 2.6.2. Intrinsic Fluorescence Measurements

Intrinsic fluorescence was measured by a Thermo Varioskan Flash 3001-1420 [21]. All samples were diluted to 1 mg/mL. The intrinsic fluorescence emission was scanned from 300 nm to 400 nm at a 280 nm excitation.

#### 2.6.3. Fourier Transform Infrared Spectroscopy (FTIR)

Secondary structure was measured by a Thermo Nicolet Nexus FTIR (Thermo Scientific, Waltham, MA, USA). One mg of lyophilized sample and 100 mg of potassium bromide were thoroughly mixed, grinded, and then placed on the ATR crystal. The number of scans was 64, the resolution was set as 4 cm^−1^. Amide I band (1700 and 1600 cm^−1^) was selected as the targeted region. Omnic (version 8.0, Thermo Nicolet Corp) and peakfit (version 4.12, AISN Software Inc.) software were employed to analyze the content of each secondary structure. Three replicates were carried out.

### 2.7. Interactions between Proteins

Interactions between proteins were determined by a previous protocol with minor modifications [22]. In order to ascertain the kind of interactions between proteins, e.g., ionic bond, hydrogen bond and hydrophobic forces, the samples were solubilized in three solutions, namely, solution A (0.6 mol/L of NaCl), solution B (0.6 mol/L of NaCl with 1.5 mol/L of urea) and solution C (0.6 mol/L of NaCl with 8 mol/L of urea). Several extraction steps were conducted by using identical conditions, including homogenization (at 5000 r/min for 2 min), extraction (at 4 °C for 1 h) and centrifugation (at 10,000× *g* for 20 min). This step was referred to as “extraction” in the following text.

Five mg of lyophilized glycinin was mixed thoroughly with 1 mL of solution A, and extraction was performed to obtain the supernatant (S1). The sediment was subsequently added to 1 mL of solution B, and extraction was again performed. The supernatant collected was referred as S2. The sediment was added to 1 mL solution C, and the supernatant was collected as S3 after extraction. Soluble protein content of S1, S2 and S3 was determined by a BCA kit (P0010, Beyotime, Shanghai, China). The analysis was conducted in triplicate.

### 2.8. Statistical Analysis

One-way analysis of variance and Duncan’s multiple comparison tests were used to determine significant differences among means (*p* < 0.05) by using SPSS version 17.0 (SPSS Inc., Chicago, IL, USA). PCA was used to observe relationships between IgE reactivity and the corresponding parameters evaluated for protein aggregation/gel and conformation changes. The graphs were generated using Origin 2019b software (OriginLab Corporation, Northampton, MA, USA).

## 3. Results and Discussion

### 3.1. pH of Fermented Glycinin

**Figure 1:**
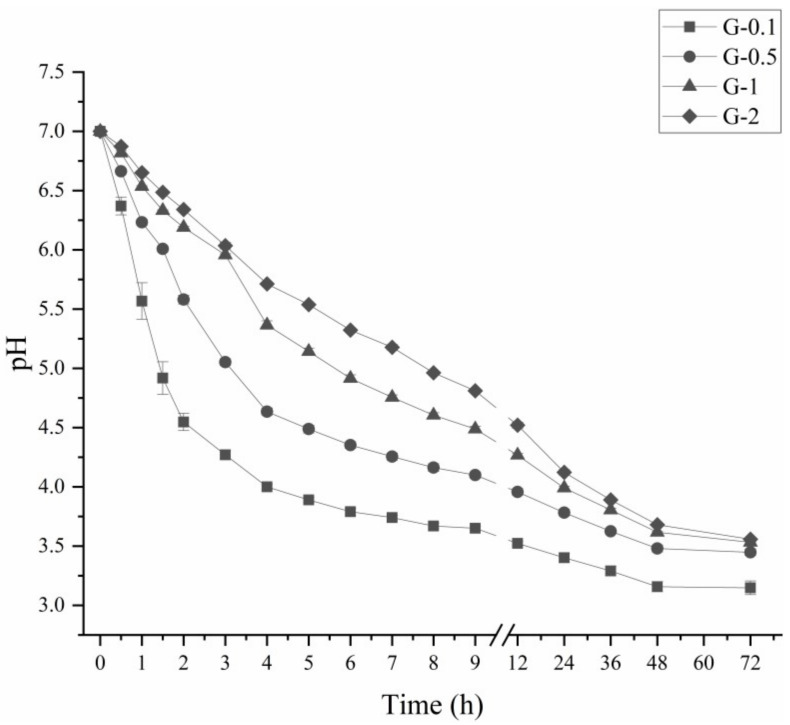
Decreases in pH of glycinins at different concentrations and fermentation time. ■: G-0.1, ●: G-0.5, ▲: G-1, ◆: G-2.

Figure 1 shows the pH of glycinin during lactic fermentation. Glycinin at different protein concentrations, namely, 0.1%, 0.5%, 1% and 2% (*w*/*v*), are abbreviated as G-0.1, G-0.5, G- 1 and G-2 hereafter. During 72 h of fermentation, the pH reduced progressively from 7.0 to 3.15–3.55 (Figure 1). This was attributed to the generation of lactic acid by lactic acid bacteria (LAB), thus lowering the pH of the glycinin solution [16]. The most significant decline was observed during the first 6 h of fermentation. The decline rate of G-0.1, G-0.5, G-1 and G-2 was 0.54, 0.44, 0.35 and 0.28 △pH/h, respectively. A higher glycinin concentration was related to a slower pH decline rate. This might correspond to the stronger buffering capacity of protein at high concentration. The decline in pH slowed down during 6–72 h of fermentation. At 72 h, G-0.1, G-0.5, G-1 and G-2 reached 3.14, 3.45, 3.53 and 3.55. According to this result, the fermentation terminal pH (FT-pH) at 7.0, 6.0, 4.5, 4.0 and 3.5 was selected in further research sampling.

### 3.2. IgE Reactivity Analysis

**Figure 2:**
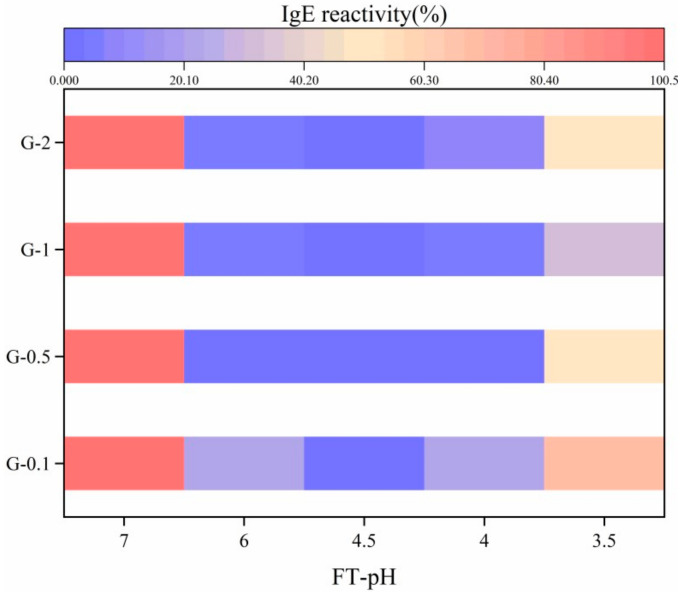
Effect of fermentation on IgE reactivity of glycinin at different concentrations.

The IgE reactivity of untreated and fermented glycinin was investigated by ELISA, and the result is presented in Figure 2. All assayed fermented glycinin, regardless of concentration and FT-pH, showed a dramatic reduction in IgE reactivity compared with untreated samples, which proved the effectiveness of L. plantarum B1-6 in of the reduction in glycinin IgE reactivity. This result confirmed our previous research, which reported L. plantarum B1-6 had the capacity to reduce the IgE reactivity of SPI [13].

The fermentation terminal pH effectively influenced IgE reactivity of glycinin. According to different terminal pH, IgE reactivity demonstrated a “U-shape” curve regardless of glycinin concentration. From FT-pH 7.0 to 4.5, IgE reactivity exerted a sharp decrease and reached the lowest value, 0.1–0.24% at FT-pH 4.5. A further decline in FT-pH induced an incomplete recovery of IgE reactivity. IgE reactivity increased to 2.70–20.24% at FT-pH 4.0 and further increased to 32.75–69.85% at FT-pH 3.5. Previous research found the IgE binding capacity of lectin decreased significantly along with the reduction in pH from 7.2 to 3.0 [14]. Due to the different types of proteins and treatment methods, our result was not completely consistent with this finding.

Glycinin concentration modulated IgE reactivity even at identical FT-pH. G-0.1 showed significantly higher IgE reactivity than the other samples throughout the fermentation process. Higher IgE reactivity was also observed for G-2 at FT-pH 6.0, 4.0 and 3.5 compared to G-0.5 and G-1. G-0.5 showed the lowest IgE reactivity at FT-pH 6.0 and 4.0, whereas significantly lower IgE reactivity was observed for G-1 at FT-pH 3.5.

Protein allergenicity is associated with many factors, such as protein structure, size and degree of protein compaction [23]. Therefore, to further explore the underlying mechanism of the immunoreactivity reduction induced by L. plantarum B1-6, the protein aggregation/gelation state, conformation and interactions between proteins were investigated to illustrate the interaction between glycinin structure and immunoreactivity.

### 3.3. Formation of Protein Gel/Aggregate

#### 3.3.1. Gelation Behavior

Table 1 summarizes the gelling behavior of glycinin at different concentrations during lactic fermentation. No gels were formed for G-0.1 and G-0.5 regardless of FT-pH, whereas gelation was initiated at FT-pH 6.0 for G-1. Double the protein concentration (G-2) did not elevate the gelation pH, but a firmer gel was observed. This indicates the gelation pH of glycinin was barely affected by its concentration but gel hardness was influenced. A similar result was observed in previous research [24]. The smallest gelling protein concentration (LGC) was 1% (*w*/*v*). This LGC was lower than those reported in some previous studies. A study showed the LGC of soy protein isolate (SPI) were 12%, 10% and 8% (*w*/*v*) at pH 3.0, 7.0 and 9.0, respectively [25]. This indicates gelation was not only related to protein concentration, but also correlated with protein type, processing technique, non-protein component and solubility [19,26]. It is presumed fermentation by LAB is more conducive to forming gels for glycinin. Gel structure was influenced by different glycinin concentrations, especially at the late fermentation stage. At FT-pH 3.5, the gel structure of G-2 was stronger and more integrate compared with G-1. The differences in gelling behavior induced by varying protein concentrations might be a result of varied protein interactions and higher structures and might further affect the immunoreactivity of glycinin.

#### 3.3.2. Particle Size Distribution

**Figure 3:**
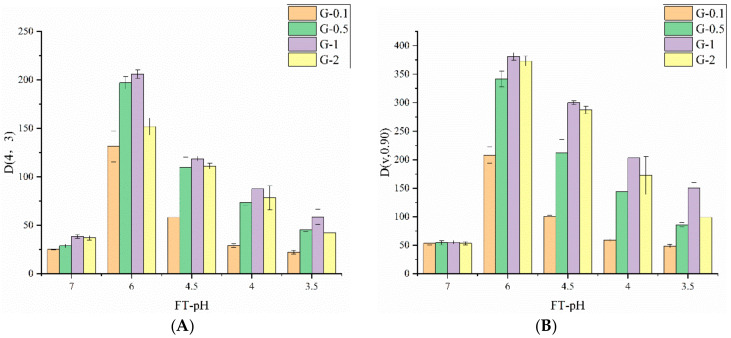
Particle size distribution for D(4,3) (**A**) and D(v,0.90) (**B**) of glycinin at different fermentation terminal pH (FT-pH). G-0.1 (orange), G-0.5 (green), G-1 (purple), G-2 (yellow).

Lactic fermentation induced the formation of glycinin aggregate/gel, resulting in changes of particle size. D(4,3) is a parameter that indicates volume-weighted mean diameter, and D(v,0.90), a parameter that reflects protein globule diameters below 90% of the volume of the proteins were chosen to monitor the change in particle size during fermentation (Figure 3) [27]. The unfermented samples demonstrated a D(4,3) value between 25.1–37 μm at FT-pH 7.0. No significant difference (*p* > 0.05) was observed between samples with different concentrations. Both D(4,3) and D(v,0.90) increased rapidly and reached the highest value (131.3–206 μm) at FT-pH 6.0, which indicates lactic fermentation triggered the formation of large protein particles. Those particles had diameters that were 5.2-, 6.8-, 5.4- and 4.1-fold greater compared with the unfermented samples of G-0.1, G-0.5, G-1 and G-2. G-0.1 was found to form the smallest particles at this stage, whereas G-1 had the largest particles. All samples demonstrated smaller particles when fermentation progressed to FT-pH 4.5 or lower, which indicates the occurrence of dynamic depolymerization of protein particles. The lowest D(4,3) value was observed for G-0.1 compared to the other investigated concentrations, which decreased to 58.10, 28.97 and 22.23 μm at FT-pH 4.5, 4 and 3.5, respectively, whereas G-1 exerted the greatest particles, with the values of 118.67, 87.53 and 58.63μm.

#### 3.3.3. SDS-PAGE

**Figure 4:**
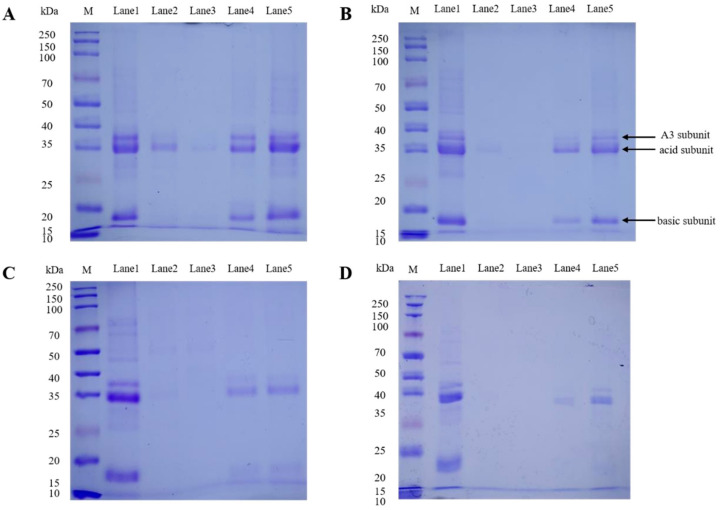
SDS-PAGE profiles of fermented glycinin at different FT-pH. (**A**) G-0.1, (**B**) G-0.5, (**C**) G-1 and (**D**) G-2. Lanes 1-5 represent FT-pH 7.0, 6.0, 4.5, 4.0 and 3.5, respectively. Subunits of glycinin (A3 subunit, acid subunit and basic subunit) are indicated by arrows.

SDS-PAGE was conducted to further investigate the protein profile of untreated and fermented glycinin (Figure 4). Unfermented glycinin exerted three subunits—11S A3, acidic and basic subunits (Lane 1, Figure 4). All fermented samples featured the rapid disappearance of basic and A3 subunits, whereas the acidic subunit remained 0–25.54% at FT-pH 6 and further dropped to 0–5.78% at FT-pH 4.5 (Lane 2–3, Figure 4). The disappearance of bands might be due to the formation of a protein aggregate/gel matrix with lower solubility, which prohibited their entrance to the gel [28]. This indicates the A3 and basic subunits might be the preferred subunits in the formation of protein aggregate (G-0.1 and G-0.5) or gel (G-1 and G-2). The acidic subunit, however, seemed to be the less preferred subunit and was partially trapped in the protein aggregates/gel matrix, especially at higher glycinin concentrations (G-0.5, G-1 and G-2) (Lane 2, Figure 4). Further fermentation allowed the bands to gradually reappear on the gel (Lanes 4–5, Figure 4). At FT-pH 4.0 or lower, all three subunits were gradually observed on the gel for G-0.1 and G-0.5 (Lane 4, Figure 4A,B), whereas G-1 and G-2 mainly demonstrated the acidic subunit (Lane 4, Figure 4C,D). This indicates the acidic subunit was more easily released from the gel/aggregation matrix compared to basic/A3 subunits. The low glycinin concentration had a stronger capacity to release the previously mentioned protein subunits from the gel/aggregation matrix.

#### 3.3.4. Discussion on the Correlation between IgE Reactivity and Formation of Protein Gel/Aggregate

LAB fermentation allowed protein gelation or aggregation to be initiated at FT-pH 6.0. Starting from this pH, glycinin was observed forming greater particles. Most the subunits, especially basic and A3 subunits, were involved in the formation of protein gel/aggregation, which is supported by the evidence that those bands disappeared rapidly on the SDS-PAGE gels. The basic and A3 subunits might be the preferred subunits for the formation of protein gel/aggregate. Ringgenberg et al. reported glycinin A3 and basic subunits were the first involved subunits in an acid induced-gel network [24]. Those aggregates/gel trapped protein subunits—especially the basic and A3 subunits—and might bury the corresponding epitopes, leading to a significantly lower IgE reactivity at FT-pH 6.0. A previous study suggested lactic fermentation might lead to a combined effect of proteolysis and acid-induced protein denaturation that allows the modification of the conformational epitopes in the protein, resulting in a total reduction in IgE binding capability [16]. At FT-pH 4.5, the further disappearance of protein bands was found on SDS-PAGE, but decreasing particle size was observed, indicating the dissociation of protein particles. This conformed to a previous study, which found the dissociation of protein particles when the pH decreased from 5.5 to 4.5 during whey protein gel formation [29]. The dissociation of protein particles was considered progress that took place to form a more uniform matrix. It is presumed this rearrangement might favor burying the linear epitopes or destroying the conformational epitopes, resulting in a further decrease in IgE reactivity. Similar results were found for the dissociation of lectin tetramers induced by low pH treatment and a decrease in IgE binding capacity [14]. At FT-pH 4.0 or lower, decreasing particle size and the recovery of protein bands at SDS-PAGE were observed. This suggests that the particles dissociated into smaller ones, and some subunits were released from the gel/aggregation matrix. This progress might lead to a looser structure, thus allowing the exposition of epitopes to the exterior environment, which might further lead to a higher IgE reactivity.

Apart from FT-pH, different glycinin concentrations exerted an important effect on the progress of gel/aggregation formation. Fermented glycinin at an extremely low concentration (G-0.1) formed smaller particles, and fewer subunits were trapped in protein aggregates, as indicated by both DLS and SDS-PAGE results. These observations might allow the exposition of epitopes and easier IgE-binding, which correspond to overall higher IgE reactivity. The higher IgE reactivity of G-0.1 was further ascribed to the higher level of free acidic subunit since significantly higher band intensity was shown on the SDS-PAGE pattern.

Previous studies confirmed acidic subunit had stronger antigenicity than that of the basic subunit [12,30]. Another investigation on E. faecalis VB34 fermented soymilk suggested that a complete disappearance of the glycinin acidic subunit probably contributes to the reduction in IgE reactivity [11]. G-0.5 and G-1 formed greater protein particles and had a similar SDS-PAGE pattern during fermentation, which resulted in adequately low IgE reactivity. The result that G-0.5 and G-1 had comparable low IgE reactivity at FT-pH 4.0–6.0 indicates that the formation of self-supporting gel barely affected immunoreactivity, although the LGC of glycinin was 1%. G-1 at FT-pH 3.5 showed a significantly lower level of the free acidic subunit compared with G-0.1, G-0.5 and G-2, which might contribute to the low IgE reactivity at this point. Improving glycinin concentration to 2% (*w*/*v*) resulted in a firmer gel and fainter bands on SDS-PAGE gels, indicating that most of the subunits were involved in the formation of protein gel. However, protein particles were smaller for G-2 accompanied by a relatively higher IgE reactivity, compared with G-0.5 and G-1. The results indicate gelation might not always favor a reduction in immunoreactivity, and protein concentration is a critical parameter in the preparation of hypoallergic soy-based foodstuffs.

### 3.4. Conformational Changes

#### 3.4.1. Surface Hydrophobicity

**Figure 5:**
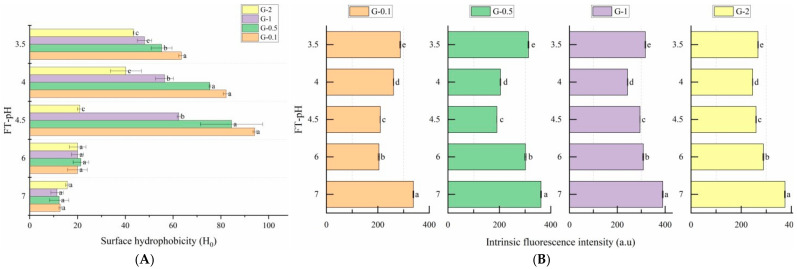
Change in surface hydrophobicity (**A**) and intrinsic fluorescence intensity (**B**) of glycinin at different FT-pH. G-0.1 (orange), G-0.5 (green), G-1 (purple), G-2 (yellow). Ho: surface hydrophobicity. a.u.: arbitrary unit. The significant difference (*p* < 0.05) between the samples collected at the same FT-pH (**A**) or at the same concentration (**B**) is denoted by lower-case letters (a–e) on the right side of bars.

Surface hydrophobicity indicates the quantity of hydrophobic amino acids residues on the surface of a protein, which is an important indicator of protein tertiary structure [31]. L. plantarum B1-6 fermentation allowed the elevation of glycinin surface hydrophobicity when FT-pH dropped to 4.5 for all investigated concentrations except G-2 (Figure 5A). The three groups of samples, namely, G-0.1, G-0.5 and G-1, demonstrated surface hydrophobicity peaked at FT-pH 4.5, at 7.39-, 6.84-, 5.46- and 1.33-fold greater compared with the untreated sample. However, the hydrophobicity of G-2 rose constantly during the fermentation process and reached a value 2.96-fold greater compared with the untreated sample at the end of fermentation. It is interesting that glycinin at higher concentrations tended to exert lower surface hydrophobicity regardless of FT-pH. It is presumed lactic fermentation induces the unfolding of protein’s native structure, leading to the exposure of hydrophobic amino acid residues to the exterior of the protein molecules [31]. It seems that samples with a higher glycinin concentration exerted less intensive unfolding of the protein structure.

#### 3.4.2. Intrinsic Fluorescence

Intrinsic fluorescence is another indicator of protein tertiary structure contributed by the fluorescence emission spectrum derived from tryptophan (Trp), tyrosine (Tyr) and phenylalanine (Phe) [32]. The fluorescence emission spectrum at 280 nm is mainly due to the existence of Trp and Tyr residues. The data (Figure 5B) show that all fermented samples suffered a significant decrease in fluorescence intensity, but no noticeable shift was observed in the wavelength of the maximum fluorescent emission. This indicates that the fermentation promoting Trp/Tyr residues shifted from an interior hydrophobic environment to an exterior hydrophilic environment [32]. This was probably related to protein unfolding induced by the deprotonation of the neighboring basic amino acids caused by a pH reduction [14]. The formation of protein gel/aggregation might further reduce the fluorescence intensity. Starting from FT-pH 6.0, the fluorescence intensity experienced steady climbing for G-0.1, whereas G-0.5, G-1 and G-2 had the lowest fluorescence intensity at FT-pH 4.5, 4.0, and 4.0, respectively. The repeated increase of fluorescence intensity was probably due to the refolding of denatured proteins and destruction of the protein gel/aggregation structure, and the initiation of this progress varied depending on glycinin concentration. It seems that the higher glycinin concentration allowed a postponed structure refolding initiated at a lower FT-pH.

#### 3.4.3. FTIR

**Figure 6:**
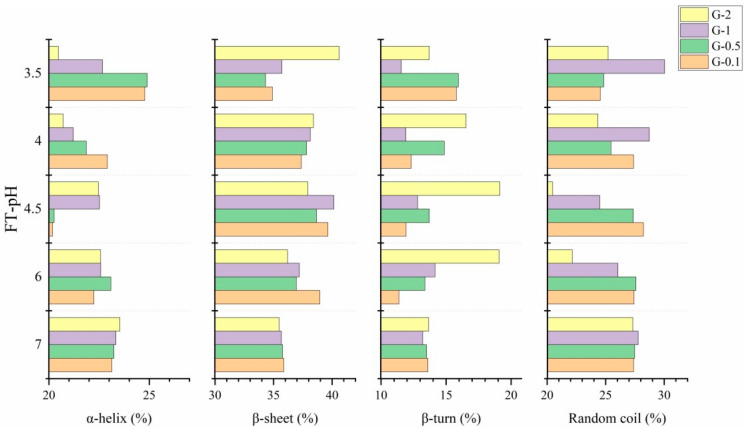
Change in secondary structures (α-helix, β-sheet, β-turn and random coil) during lactic fermentation under different concentrations. x axis: samples collected at different FT-pH values; y axis: four secondary structures (α-helix, β-sheet, β-turn and random coil). G-0.1 (orange), G-0.5 (green), G-1 (purple), G-2 (yellow).

FTIR was used to determine the secondary structure of untreated and fermented glycinin (Figure 6). The assignments of the secondary structure were given according to a previous study that assigned amide I range (1600–1700 cm^−1^) into four different secondary structures, namely, α-helix (1650–1660 cm^−1^), β-sheet (1620–1640 cm^−1^ and 1670–1680 cm^−1^), β-turn (1660–1670 cm^−1^ and 1680–1695 cm^−1^) and random coil (1640–1650 cm^−1^) [33,34]. The results show that both untreated (FT-pH 7.0) and fermented glycinin consists of four structures.

Fermentation induced an overall reduction in α-helix structure. The lowest value (20.18–22.52%) was obtained at FT-pH 4.5 for G-0.1, G-0.5 and G-1 and at FT-pH 3.5 for G-2 (20.46%). The decreases in α-helix might indicate the process of unfolding and denaturation [21]. The β-sheet increased at FT-pH 6.0 and reached the highest value (38.66–40.16%) at FT-pH 4.5 for G-0.1, G-0.5 and G-1, whereas G-2 increased constantly to 40.60% at FT-pH 3.5. The disappearance of the α-helix might be due to its transformation into β-sheet, which was suggested as an important conformational transition during protein aggregation [35]. A previous study suggested the unfolding of α-helix and the formation of β-sheets might be favored in the gelation of glycinin [36]. Zhao et al. (2013) suggested the hydrolysis of soy protein leads to the transformation of an α-helix into a β-sheet [32]. Glycinin concentration affected the secondary structures of fermented samples. A significantly higher proportion of β-turn and lower random coil content were observed for G-2 at both FT-pH 6.0 and 4.5, which demonstrated a unique secondary structure. This phenomenon might correlate to the maintenance of protein structures in an ordered state [12], indicating a more compact gel structure of G-2.

#### 3.4.4. Discussion on the Correlation between IgE Reactivity and Protein Conformation

Previous studies predicted 29 linear epitopes located on different subunits of glycinin, most of them located on the surface and hydrophilic areas [37,38]. Conformational changes of glycinin might allow the alternation of those epitopes correspondingly. The results of conformational investigations indicate the secondary and tertiary structures were altered significantly during fermentation, especially at FT-pH 6.0 and 4.5-, at which the lowest value of IgE reactivity was obtained. At FT-pH 6.0, the surface hydrophobicity of fermented glycinin reached the highest value, and intrinsic fluorescence intensity decreased, accompanied by a significant decrease in α-helix structure and the increase of β-sheet structure. These changes indicate the unfolding of glycinin structure. A previous study proposed the opening of α-helix structure led to the exposure of hydrophobic and sulfhydryl groups, which indicates the initiation of the soy protein aggregation [39]. Some linear epitopes (i.e., 217–235) that were reported to be laid in the α-helix structures might be disrupted at this fermentation stage, thus resulting in lower immunoreactivity [7,40]. The opening of α-helix might also destroy some conformational epitopes and bury some epitopes inside the aggregated proteins [41].

The tertiary structure of glycinin was extensively altered during fermentation. The increase in surface hydrophobicity and decrease in intrinsic fluorescence intensity indicates the unfolding of glycinin. Among 36 Trp/Tyr residues on glycinin, some of them were involved in the constitution of epitopes. There were four epitopes in total (136–153, 83–97, 177–191, 189–203) located on the acid subunit and four epitopes (337–351, 345–359, 358–372, 367–381) located on the basic subunit containing Trp/Tyr residues. Those epitopes might be altered during fermentation, hindering their interactions with Ig E [37,42]. The opening of the tertiary structure might lead the epitopes located on the surface of proteins to be buried and hidden in the structure, such as the epitopes mentioned previously or those epitopes located on the surface of glycinin subunits (i.e., 121–129, 130–141, 214–225, 256–261 and 283–291) [7]. This hypothesis was confirmed by a previous study, which observed a reduction in immunoreactivity in glycinin after the treatment of microbial transglutaminase (MTG) combined with heat treatment; the authors partly attributed the decrement of immunoreactivity to the enhanced surface hydrophobicity [12]. Another research also indicated IgE reactivity reduction correlated to the decrease in intrinsic fluorescence intensity in acid-treated lectin and suggested the unfolding of protein structures might be correlated to a reduction in immunoreactivity [14]. As fermentation progresses, varying degrees of renaturation of glycinin were observed. During this process, the secondary structure demonstrated a major increase α-helix and a decrease in β-sheet structure at FT-pH 4.0 or lower. In addition, lower surface hydrophobicity and higher intrinsic fluorescence intensity at FT-pH 4.5 or lower were shown. Restoration of the tertiary structure was accompanied by partial recovery of IgE reactivity. It is presumed the re-exposition of epitopes is due to the restoration of glycinin structures.

### 3.5. Interactions between Proteins

**Figure 7:**
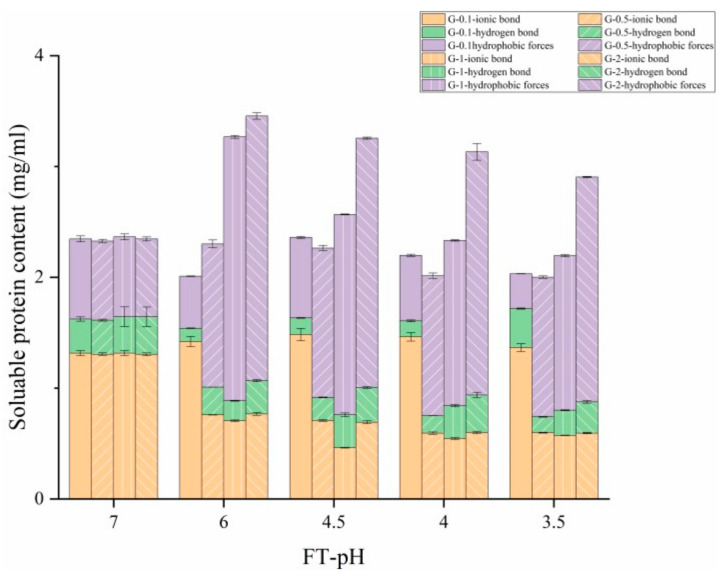
Influence of lactic fermentation on interaction among proteins (ionic bond, hydrogen bond and hydrophobic forces) of glycinin at different concentrations.

Figure 7 represents the proportions of protein fractions that interacted with the ionic bond, hydrogen bond and hydrophobic forces. The initiation of fermentation allowed a total enhancement of interactions between proteins, which peaked at FT-pH 6.0 for G-0.5, G-1 and G-2. G-0.1; however, the highest value was demonstrated at FT-pH 4.5. Protein interactions reached the highest point at FT-pH 6.0 for most of the investigated samples, probably due to the aggregation or gelation of glycinin [26,43]. This was consistent with the result of the gelation behavior and greatest particle size obtained at this FT-pH. A decrease in protein interactions indicates a looser structure at FT-pH 4.5, 4.0 and 3.5. In addition, samples with higher glycinin concentration elicited more intensive interactions between proteins, suggesting a higher requirement of interactive forces was needed to maintain their structures.

Untreated glycinin was found to be mainly maintained by ionic bonds. At FT-pH 6.0, a major shift of dominant interaction from ionic bonds to hydrophobic forces was observed for most of the investigated samples. Ionic bond interaction reached the lowest value at FT-pH 4.5 to 4.0 for G-0.5, G-1 and G-2. It was presumed that decreasing electrostatic repulsion and net charge caused by a pH reduction approaching the isoelectric point of glycinin might contribute to this reduction [44]. Samples with higher glycinin concentration exerted stronger hydrophobic interactions. Fermented G-0.1 showed a very different pattern. Those samples predominantly interacted with ionic bonds followed by hydrophobic forces. It could be concluded that hydrophobic forces were the main forces maintaining the glycinin gel structure induced by lactic fermentation, whereas ionic bonds and hydrogen bonds play complementary roles. This is consistent with a previous study [45].

### 3.6. Principal Components Analysis (PCA)

**Figure 8:**
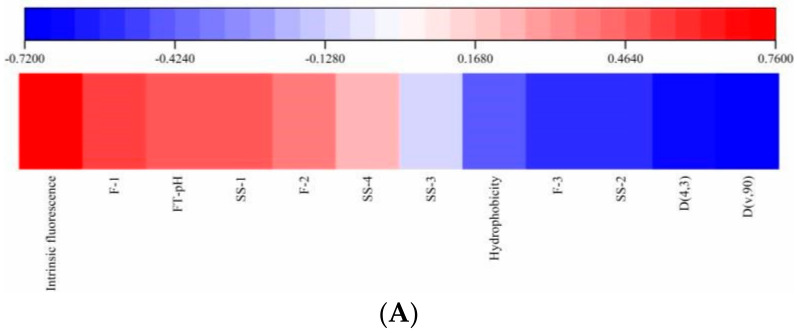

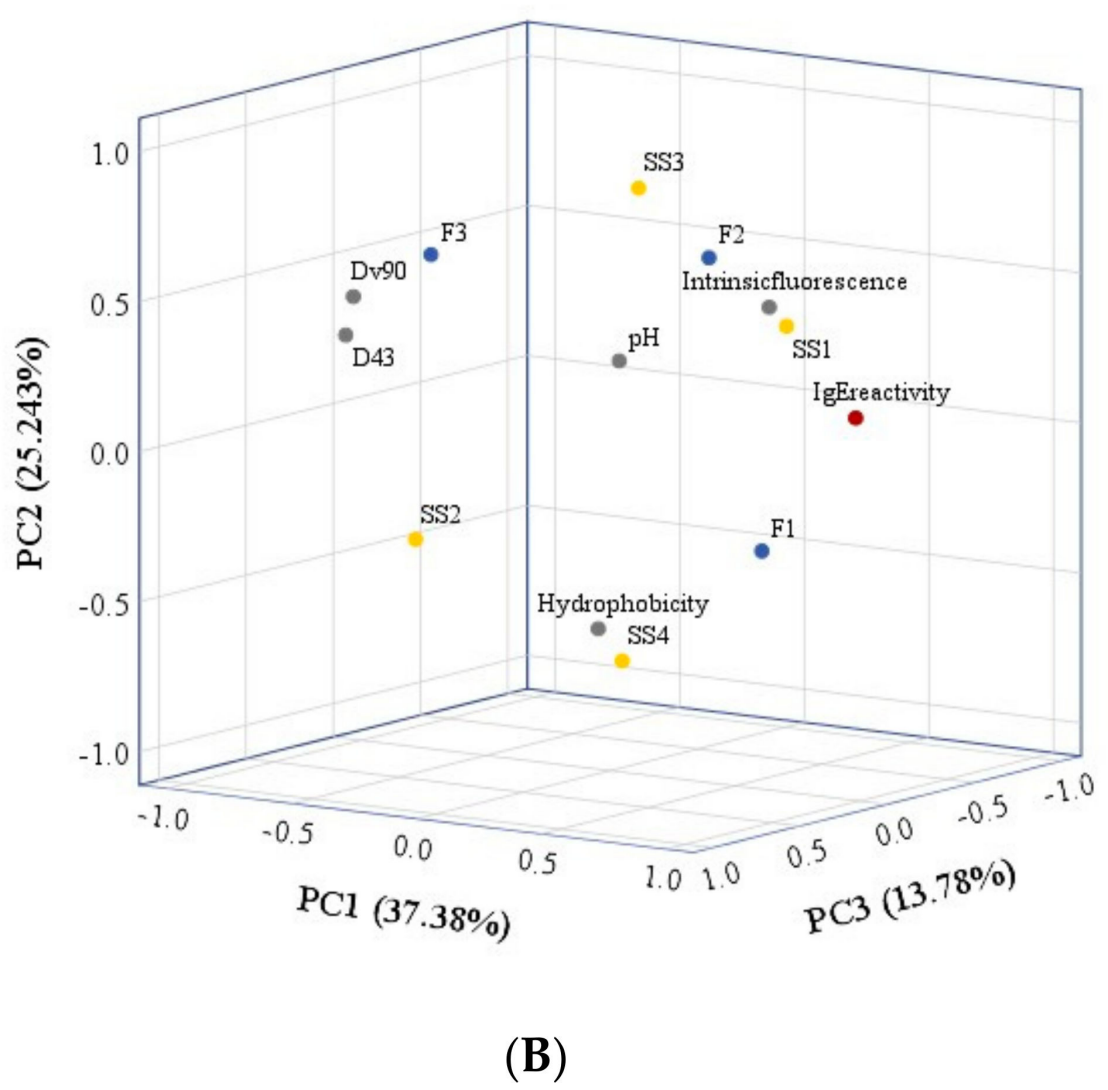
Principal component analysis (PCA) loading plot (**A**) and correlation coefficient (**B**) between IgE reactivity and conformational parameters. D43: D(4,3), Dv90: D(v,90), SS: secondary structure (SS−1: αhelix; SS−2: β-sheet; SS−3: β-turn; SS−4: random coil), F: interaction force (F−1: ionic bond; F−2: hydrogen bond; F−3: hydrophobic forces).

PCA was conducted to identify the relationship between IgE reactivity, reduction against FT-pH, particle size and conformational index (Figure 8). Four principal components (PC), PC1-4, represented 37.38%, 25.24%, 13.78% and 9.33%, respectively, of a variable majority (85.73%). IgE reactivity (0.933, 0.147, −0.074, 0.230) was closely correlated with intrinsic fluorescence intensity (0.717, 0.522, 0.113, 0.070), SS1 (α-helix) (0.595, 0.407, −0.160, −0.599), F1 (ionic bond) (0.722, −0.283, 0.162, −0.094), and pH (0.496, 0.400, 0.657, 0.133), indicating fermentation-induced conformational changes greatly affected the glycinin IgE reactivity. D(4,3) (−0.651, 0.359, 0.585, −0.268) and D[v,0.90] (−0.740, 0.454, 0.415, −0.190) showed a negative correlation with IgE reactivity, demonstrating that glycinin aggregated into large particles favored a reduction in IgE reactivity. Meinlschmidt suggested protein aggregation had a major contribution on IgE reactivity reduction [16], which was confirmed by our investigation. In addition, hydrophobicity (−0.351, −0.736, −0.433, −0.176), F-3 (hydrophobic forces) (−0.752, 0.526, −0.046, 0.174) and SS-2 (β-sheet) (−0.661, −0.383, 0.172, 0.490) showed a negative correlation with IgE reactivity, whereas SS-3 (β-turn) (−0.239, 0.733, −0.505, −0.101) and SS-4 (0.390, −0.635, 0.488, 0.045) were less correlated with IgE reactivity. These structural changes, overall, indicate an opening of the protein structures, which might lead the epitopes located on the surface of proteins to be buried and hidden in the structure and the destruction of some conformational epitopes. Previous research also found the unfolding of protein secondary and tertiary structures resulted in reduced immunoreactivity [46].

## 4. Conclusions

Fermentation with L. plantarum B1-6 exerted potent capacities in the reduction in IgE reactivity of glycinin to 0.10%–69.85%. L. plantarum B1-6-induced protein aggregation/gelation resulted in the evolvement of larger particles, especially G-1, which has the greatest particles (58.63–206 μm). Most subunits were involved in protein aggregates/gels, especially A3 and basic subunits. Conformational changes in fermented glycinin were proved on secondary and tertiary structures, especially at fermentation terminal pH (FT–pH) 4.5, the lowest point of IgE reactivity (0.1–0.24%). At this point, a dramatic increase in surface hydrophobicity, a significant reduction in intrinsic fluorescence intensity, and an α-helix transformed into β-sheet structure were observed. Additionally, increased interactions between proteins were also found.

The results indicate that reduction in IgE reactivity by lactic fermentation was related to the formation of protein gel/aggregates and the opening of protein higher structure. Linear epitopes located on the surface of glycinin (i.e., 121–129, 130–141, 214–225, 256–261 and 283–291) might be buried and/or conformational epitope might be destroyed during this process, thus contributing to the overall reduction in immunoreactivity. The mechanism of aggregation and/or conformation changes was FT-pH and concentration-dependent. Performing further studies focusing on in vivo and in vitro experiments to explore the allergenicity and digestibility changes, especially glycinin at FT–pH 4.5 and 3.5, would be interesting.

## Figures and Tables

**Table 1:** Gelling behavior of different concentrations of glycinin proteins during lactic fermentation.

Samples	pH 7.0	pH 6.0	pH 4.5	pH 4.0	pH 3.5
G-0.1	⊝⊝	⊝⊝	⊝⊝	⊝⊝	⊝⊝
G-0.5	⊝⊝	⊝±	⊝±	⊝±	⊝±
G-1	⊝⊝	√√	√√	√√	√±
G-2	⊝⊝	√√	√√	√√	√√

⊝ No gelation; ± Weak gel; √ Gelation; √√ Firm gel; √ Smallest gelling protein concentration (LGC).

## Data Availability

The data presented in this study are available on request from the corresponding author.
